# An endorectal ultrasound-based radiomics signature for preoperative prediction of lymphovascular invasion of rectal cancer

**DOI:** 10.1186/s12880-022-00813-6

**Published:** 2022-05-10

**Authors:** Yu-quan Wu, Rui-zhi Gao, Peng Lin, Rong Wen, Hai-yuan Li, Mei-yan Mou, Feng-huan Chen, Fen Huang, Wei-jie Zhou, Hong Yang, Yun He, Ji Wu

**Affiliations:** 1 Department of Medical Ultrasound, The First Affiliated Hospital of Guangxi Medical University, 6 Shuangyong Road, Nanning, Guangxi Zhuang Autonomous Region People’s Republic of China; 2 The Second Clinical Medical College, Guangxi Medical University, No. 22 Shuangyong Road, Nanning, Guangxi Zhuang Autonomous Region People’s Republic of China

**Keywords:** Ultrasound, Radiomics, Model, Rectal cancer, Lymphovascular invasion

## Abstract

**Objective:**

To investigate whether radiomics based on ultrasound images can predict lymphovascular invasion (LVI) of rectal cancer (RC) before surgery.

**Methods:**

A total of 203 patients with RC were enrolled retrospectively, and they were divided into a training set (143 patients) and a validation set (60 patients). We extracted the radiomic features from the largest gray ultrasound image of the RC lesion. The intraclass correlation coefficient (ICC) was applied to test the repeatability of the radiomic features. The least absolute shrinkage and selection operator (LASSO) was used to reduce the data dimension and select significant features. Logistic regression (LR) analysis was applied to establish the radiomics model. The receiver operating characteristic (ROC) curve, calibration curve, and decision curve analysis (DCA) were used to evaluate the comprehensive performance of the model.

**Results:**

Among the 203 patients, 33 (16.7%) were LVI positive and 170 (83.7%) were LVI negative. A total of 5350 (90.1%) radiomic features with ICC values of ≥ 0.75 were reported, which were subsequently subjected to hypothesis testing and LASSO regression dimension reduction analysis. Finally, 15 selected features were used to construct the radiomics model. The area under the curve (AUC) of the training set was 0.849, and the AUC of the validation set was 0.781. The calibration curve indicated that the radiomics model had good calibration, and DCA demonstrated that the model had clinical benefits.

**Conclusion:**

The proposed endorectal ultrasound-based radiomics model has the potential to predict LVI preoperatively in RC.

**Supplementary Information:**

The online version contains supplementary material available at 10.1186/s12880-022-00813-6.

## Background

According to global cancer statistics in 2020, rectal cancer (RC) is a major threat to humans. The RC has the third-highest recorded incidence of cancer, which is listed after lung cancer and breast cancer, and the cancer-related mortality rate of RC is ranked second after lung cancer [1]. Early detection and diagnosis is crucial to effectively treat RC. Unfortunately, most patients are diagnosed with locally advanced rectal cancer (LARC), which means RC with stage T3 or T4 [2]. The standard treatment option for LARC is neoadjuvant chemoradiotherapy followed by total mesorectal excision (TME) [3, 4]. However, even though patients with LARC have already received neoadjuvant chemoradiotherapy, the 5-year overall survival rate is only 66.3% [5].

The poor prognosis of RC is related to various factors, including lesion size, stage, lymphovascular invasion (LVI) status, tumor marker status, immune indicators that are positive, RC’s sensitivity to radiotherapy and chemotherapy, and so on [6].

Previous studies have shown that vascular invasion is an independent risk factor for poor prognosis of RC [7, 8]. Now, the LVI status can be obtained by only postoperative pathology, which therefore cannot guide whether the patient should be treated more aggressively preoperatively. Therefore, preoperative prediction of LVI in RC is helpful for clinical decision-making.

There are numerous means of preoperative examination for RC. Colonoscopy can observe the general surface of the mass and perform the biopsy; however, the disadvantage is that the depth of infiltration and extraintestinal invasion could not be observed. Magnetic resonance imaging (MRI), especially high-resolution MRI, is the first choice for preoperative imaging diagnosis and postoperative follow-up review, which can accurately perform tumor node matastasis (TNM) staging, but its high cost makes it difficult to repeat multiple examinations, and it is always difficult to distinguish the T2 stage from the T3 stage [9, 10]. Endoscopic ultrasound (EUC) and transrectal ultrasound (TRUS) are cheap and repeatable and can visualize structures at multiple levels of the intestinal wall, but they still do not solve the problem of the preoperative prediction of LVI [11, 12]. To sum up, to optimize individualized clinical treatment, a method that can noninvasively and preoperatively predict the presence or absence of LVI is an urgent clinical need.

In recent years, radiomics has been introduced and has the potential to provide help for the above clinical challenges. Radiomics, using computers to excavate the data of medical images (ultrasound, CT, MR, etc.) to discover the intrinsic relationship between medical images and features, such as diagnostic localization, biological behavior, and treatment outcome of diseases, could provide a reference for clinical decision-making. Several recent reviews systematically reviewed artificial intelligence (AI) models and machine learning about radiomics based on high-resolution MRI which could be used to predict the response of chemotherapy and distant metastasis of RC, and the results showed that this novel imaging technology might be helpful to clinical decision-making [13–16]. Chinese scholars have reported that CT radiomics can predict whether lymph node metastasis exists preoperatively in RC [17], and CT radiomics and MR radiomics can predict colorectal cancer LVI [18, 19]. Also, high-resolution MR radiomics could predict peripheral nerve invasion [20] and disease-free survival [21] in RC, assessing response to treatment for RC [22, 23]. Ultrasound radiomics can predict subtypes of liver cancer [24] and determine whether lymph node metastasis exists in thyroid cancer [25]. However, to the best of our knowledge, studies of endorectal ultrasound-based radiomics in the LVI have not yet been reported. Our study aims to utilize endorectal ultrasound-based radiomics techniques to predict LVI preoperatively and noninvasively to aid in making clinical decisions.

## Materials and methods

### Subjects

The Ethics Committee of our hospital approved this retrospective study. Informed consent was waived. Patients with RC who underwent surgery in our hospital from January 2018 to February 2021, were included in this study. Inclusion criteria were the following: (1) RC that was confirmed by postoperative pathology, (2) TRUS examination that was conducted before surgery, (3) the biggest section of the RC lesion image that was clear and complete (in the case of multiple lesions, the largest lesion was to be selected), and (4) the required clinicopathological data that were complete. The exclusion criteria were as follows: (1) previous history of RC, (2) unclear diagnosis of LVI, and (3) neoadjuvant chemoradiotherapy if performed before ultrasound examination.

### Ultrasound examination methods

First, the patient was asked to lie on the examination bed with a left lateral coxa and knee bending position, take off pants, and fully expose the anus. Before the TRUS examination, a digital rectal examination (DRE) was conducted to judge whether anal stenosis exists and the distance between the lower edge of the lesion and the anus, the lesion’s size, and its position. Then, a 50-ml mixture of water and starch was instilled into the rectal cavity through the anus, and the TRUS probe was wrapped in a condom for isolation when the probe was extended into the anal canal for examination. A convex or line array probe was selected according to the lesion’s size and distance between the lower edge of the lesion and the anus. An experienced sonographer performed a comprehensive scan of the lesion to assess the lesion’s size, shape, margin, and internal echogenicity, the distance between the lower edge of the lesion and the anus, and whether muscularis, serosal layer, internal anal sphincter, and external anal sphincter invasion existed. The images were stored for future use.

### Instruments and image acquisition

Esaote MyLab Class C and General Electric Company (GE) LOGIQ E9 ultrasound diagnostic instruments were used to acquire ultrasound images of the lesions. The frequency of the probe was 6–15 MHz, and the images were recorded and saved in the Digital Imaging and Communications in Medicine (DICOM) format.

### Clinical and pathological data

Clinical and pathological data, such as the patient’s gender, age, tumor size, stage, vascular invasion, and serum CEA, CA125, CA199, CA242, CA742, etc., were collected.

### Region of interest (ROI) segmentation, feature extraction, and consistency analysis

In this study, the ROIs of the lesions were outlined using the image software application ITK-SNAP(3.8.0), which is an open source for medical image segmentation (Fig. 2) [24]. The ROIs were outlined manually by two independent radiologists (reader 1 with 5 years of experience in TRUS examination and reader 2 with 10 years). Neither radiologist knew the pathology results. Also, 50 images were randomly selected from all the images and drawn independently by two doctors to evaluate consistency between different observers. Subsequently, we performed feature extraction of the tumor ROI using Intelligent Foundry software (version 1.3, GE Company, Shanghai). This software extracted massive amounts of image features and represented them as quantitative data, which could be used to analyze the potential heterogeneity within the tumors [17]. Finally, we used consistency analysis to gain consistency between different observers, and we considered the features with consistency tests greater than 0.75 as those with high confidence and retained them for subsequent studies.

### Feature preprocessing and selection

In this study, we used the Cart package in R language (version 4.3.0) to stratify all patients in a 7:3 ratio by the computer randomization method, with 70% of patients entering the training set for model construction and 30% of patients entering the validation set for model verification. Different evaluation metrics (radiomic features) often had different scales and scale units. To increase the comparability of quantitative radiomic features, we performed Z-score normalization for quantitative features in training and validation sets, respectively. Subsequently, to select the radiomic features associated with LVI in RC from the numerous image features, we performed a hypothesis test on all features in the training set and retained the features with statistically significant significance in the hypothesis test for the multifactor regression analysis. The massive number of radiomic features constructed in the model easily led to model overfitting, and to reduce the influence of high-dimensional features on the model, we used LASSO regression for feature downscaling and tenfold cross-validation to filter the optimal subset of features to predict LVI in RC from radiomic features. Meanwhile, we performed a correlation analysis of all features to optimize the covariance effect of the subset of features. We considered a correlation coefficient of ≥ 0.8 to be a high correlation.

### Construction, evaluation, and validation of the imaging radiomics model

To construct the radiomics model, we performed multifactor logistic regression modeling using the optimal subset of features in the training set. Meanwhile, we used the area under the curve (AUC) of the receiver operating characteristic (ROC) curve of both training and validation sets to evaluate the comprehensive discrimination ability of the radiomics model. We also used calibration curves to calculate the agreement between the predicted and observed probabilities. We used decision curve analysis (DCA) to estimate the outcome of the prediction model under different threshold probabilities.

### Statistical analysis

SPSS 22.0 and R language (version 4.3.0) were used for the analysis. For statistical analysis of clinical parameters and hypothesis testing of radiomics characteristics, the chi-square test or Fisher’s exact probability method was used for statistical analysis of count data, and an independent samples t-test or Mann–Whitney U test was performed for measurement data. Means ± standard deviations were used for measures that conformed to a normal distribution and medians for those abnormal distributions. A P-value of < 0.05 was considered statistically significant.

## Results

### Clinicopathological data of the patients

**Fig. 1:**
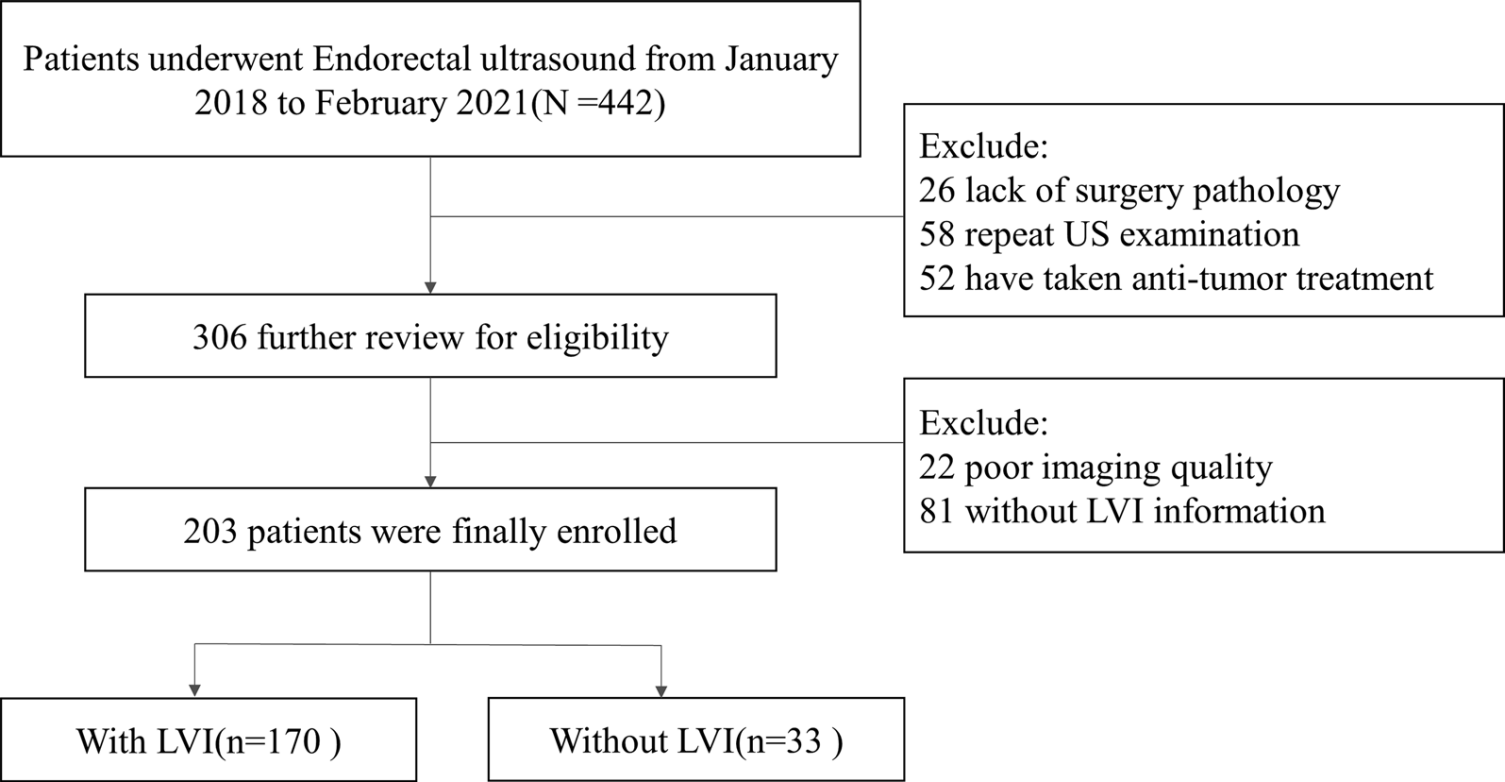
Flow chart of screening the patients with rectal cancer based on the study enroll criteria

According to the above-mentioned inclusion and exclusion criteria, 203 patients with RC were finally included (Fig. 1). The age range of the patients was 19–89 years, and the mean was 59.4 ± 12.4 years. There were 170 cases in the LVI-negative group and 33 cases in the LVI-positive group. The clinicopathological information of the patients is shown in Table 1. The differences in age, gender, maximum tumor diameter, thick diameter, ultrasound T-stage, N-stage, CEA, CA199, CA125, CA242, and CA724 between the two sets have no statistical significance, suggesting that the random grouping of these two groups is reasonable.

**Table 1:** Clinical and pathological information on rectal cancer

Variable	Training set (n = 143)	Validation set (n = 60)	*p* value
*Age*			0.101
< 60 years	63 (44.1%)	34 (56.7%)	
≥ 60 years	80 (55.9%)	26 (43.3%)	
*Sex*			0.632
Female	60 (42%)	23 (38.3%)	
Male	83 (58%)	37 (61.7%)	
*Tumor maximum diameter*			0.999
≤ 3 cm	31 (21.7%)	13 (21.7%)	
> 3 cm	112 (78.3%)	47 (78.3%)	
*Tumor thickness diameter*			0.442
≤ 1 cm	18 (12.6%)	10 (16.7%)	
> 1 cm	125 (87.4%)	50 (83.3%)	
*T stage*			0.242
T1–2	29 (20.3%)	8 (13.3%)	
T3–4	114 (79.7%)	52 (86.7%)	
*N stage*			0.558
N0	85 (59.4%)	33 (55%)	
N1–2	58 (40.6%)	27 (45%)	
*CEA*			0.213
Negative	77 (53.8%)	38 (63.3%)	
Positive	66 (46.2%)	22 (36.7%)	
*CA199*			0.824
Negative	114 (79.7%)	47 (78.3%)	
Positive	29 (20.3%)	13 (21.7%)	
*CA125*			0.588
Negative	132 (92.3%)	54 (90%)	
Positive	11 (7.7%)	6 (10%)	
*CA242*			0.871
Negative	97 (67.8%)	40 (66.7%)	
Positive	46 (32.2%)	20 (33.3%)	
*CA724*			0.709
Negative	116 (81.1%)	50 (83.3%)	
Positive	27 (18.9%)	10 (16.7%)	
*LVI*			0.465
Negative	118 (82.5%)	52 (86.7%)	
Positive	25 (17.5%)	8 (13.3%)	

### Extraction and selection of endorectal ultrasound-based radiomic features

**Fig. 2:**
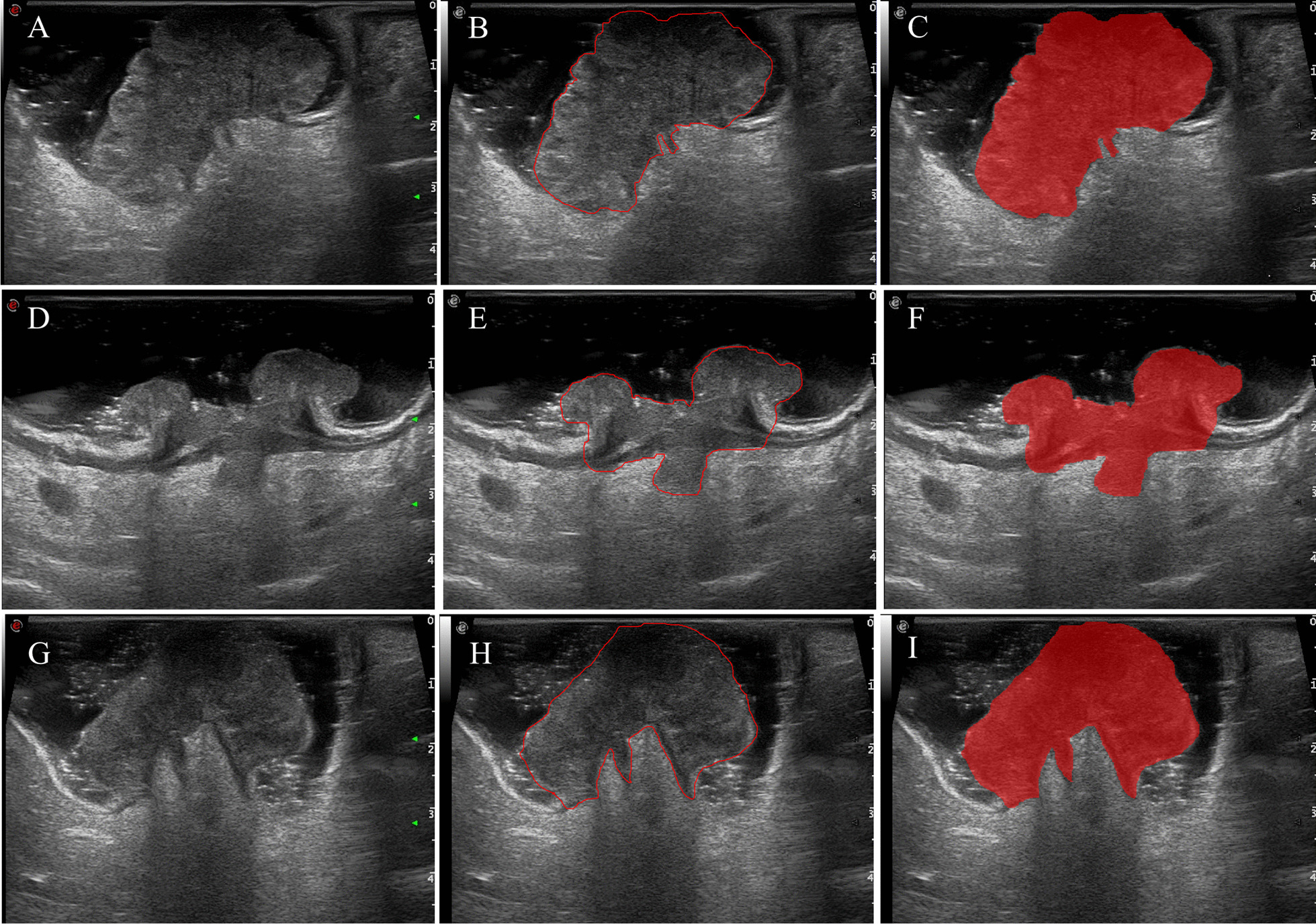
Schematic outline of the region of interest (ROI) of a rectal carcinoma. **a**, **d**, **g** The biggest section of the ultrasound image of rectal cancer. **b**, **e**, **h** The red line delineates the ROI along the edge of the lesion. **c**, **f**, **i** Schematic of the cut image. **a**, **b**, **c** show a LVI negative case. **d**, **e**, **f** show another LVI negative case. **g**, **h**, **i** show a LVI positive case

**Fig. 3:**
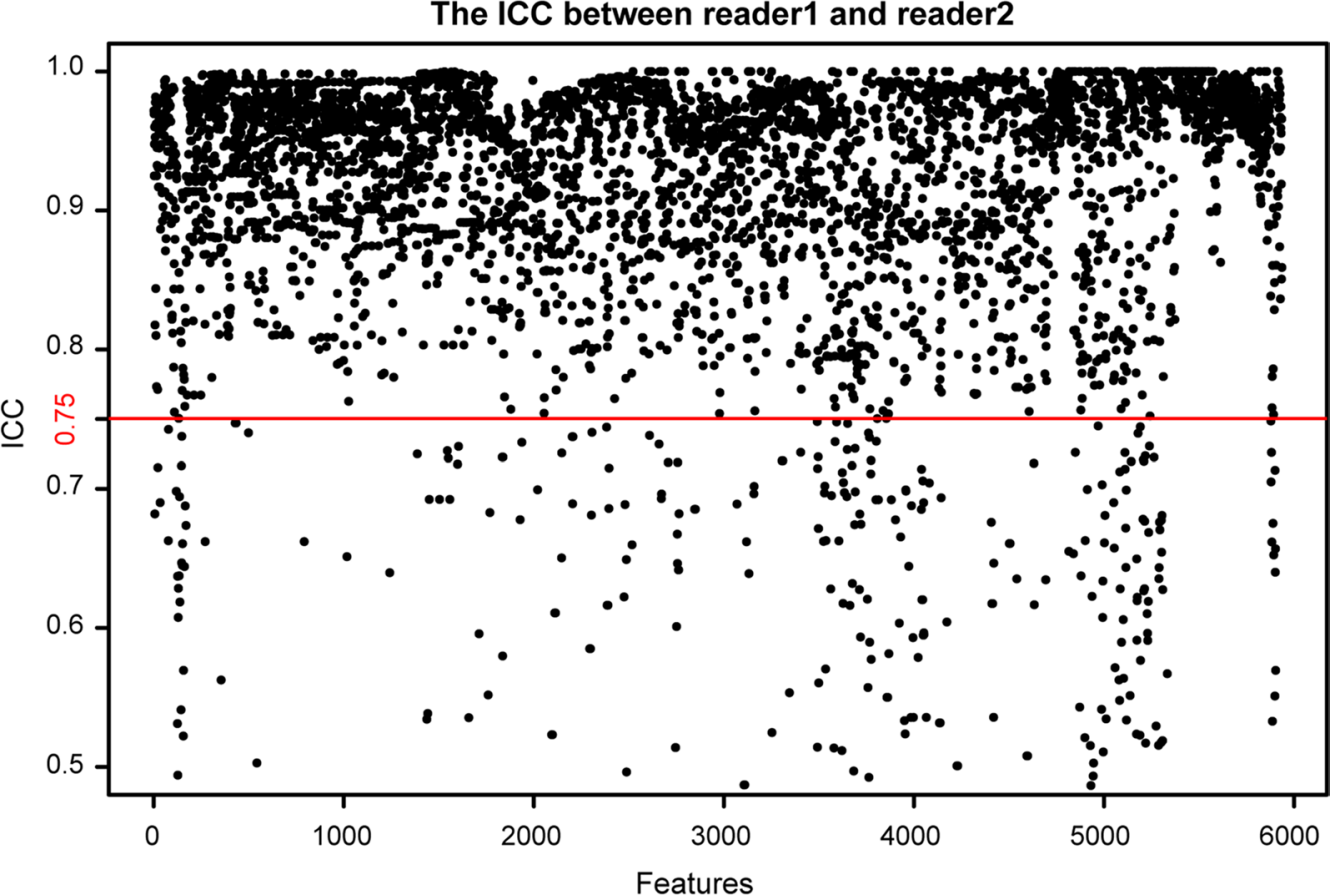
The intraclass correlation coefficient (ICC) test between readers 1 and 2. Among 5936 features, a total of 5350 (90.1%) features had ICC values of ≥ 0.75

**Fig. 4:**
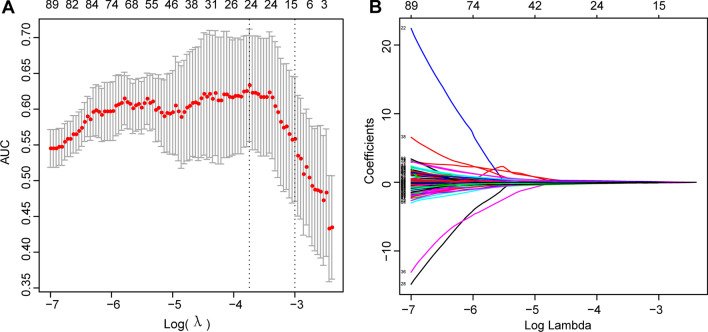
Selecting features via the least absolute shrinkage and selection operator (LASSO) logistic regression analysis. **a** Using tenfold cross-validation to select the tuning parameter (λ) in the LASSO model via minimum criteria. The correlation between the AUC curve and log(λ) was plotted in this chart. Dotted vertical lines were drawn at optimal values. **b** LASSO coefficient profiles of 179 features. The coefficient profile was plotted against the log (λ) sequence. After the tenfold cross-validation, the optimal λ resulted in 15 nonzero coefficients

**Fig. 5:**
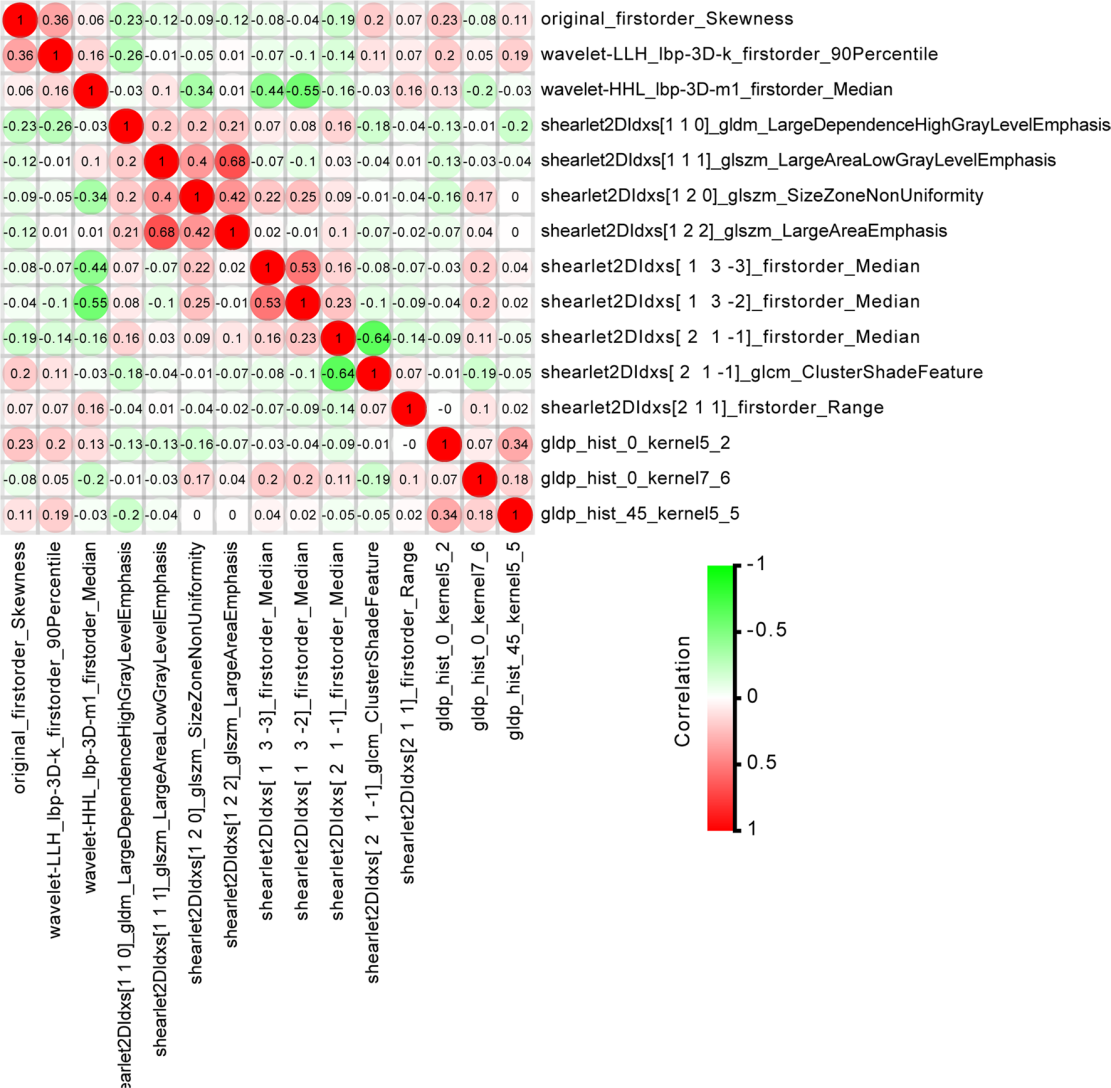
Correlation of radiomic features associated with lymphovascular invasion. The degree of correlation between various features is shown in different shades of color. Red indicates that they are positively correlated, while green indicates that they are negatively correlated

The Intelligence Foundry software was used to extract the features of the outlined ROIs (Fig. 2), and a total of 5936 image features were obtained. The radiomic features were listed as follows: (1) 122 original features (first-order statistics, texture classes, shape descriptors, etc.); (2) 48 intra-perinodular textural transitions (ipris); (3) 432 wavelets + local binary patterns (LBPs); (4) 1170 co-occurrence of local anisotropic gradient orientations (CoLIAGe); (5) 1080 Gabors; (6) 2944 shearlets; (7) 60 wavelet-based improved local binary patterns (WILBPs); and (8) 80 phased congruency-based local binary patterns (PLBPs). The ICC test results showed 5350 (90.1%) radiomic features with ICC coefficients of ≥ 0.75 (Fig. 3), which were considered stable and used for subsequent analysis. Through hypothesis testing, we found a total of 179 radiomic features associated with colorectal cancer LVI in the training set. A total of 15 features were used as the optimal subset for predicting LVI in RC after LASSO regression combined with tenfold cross-validation (Fig. 4). The correlation analysis showed that the maximum correlation coefficient between the radiomic features of the optimal feature subset was 0.68 (Fig. 5).

### Radiomics model establishment and efficacy assessment

**Fig. 6:**
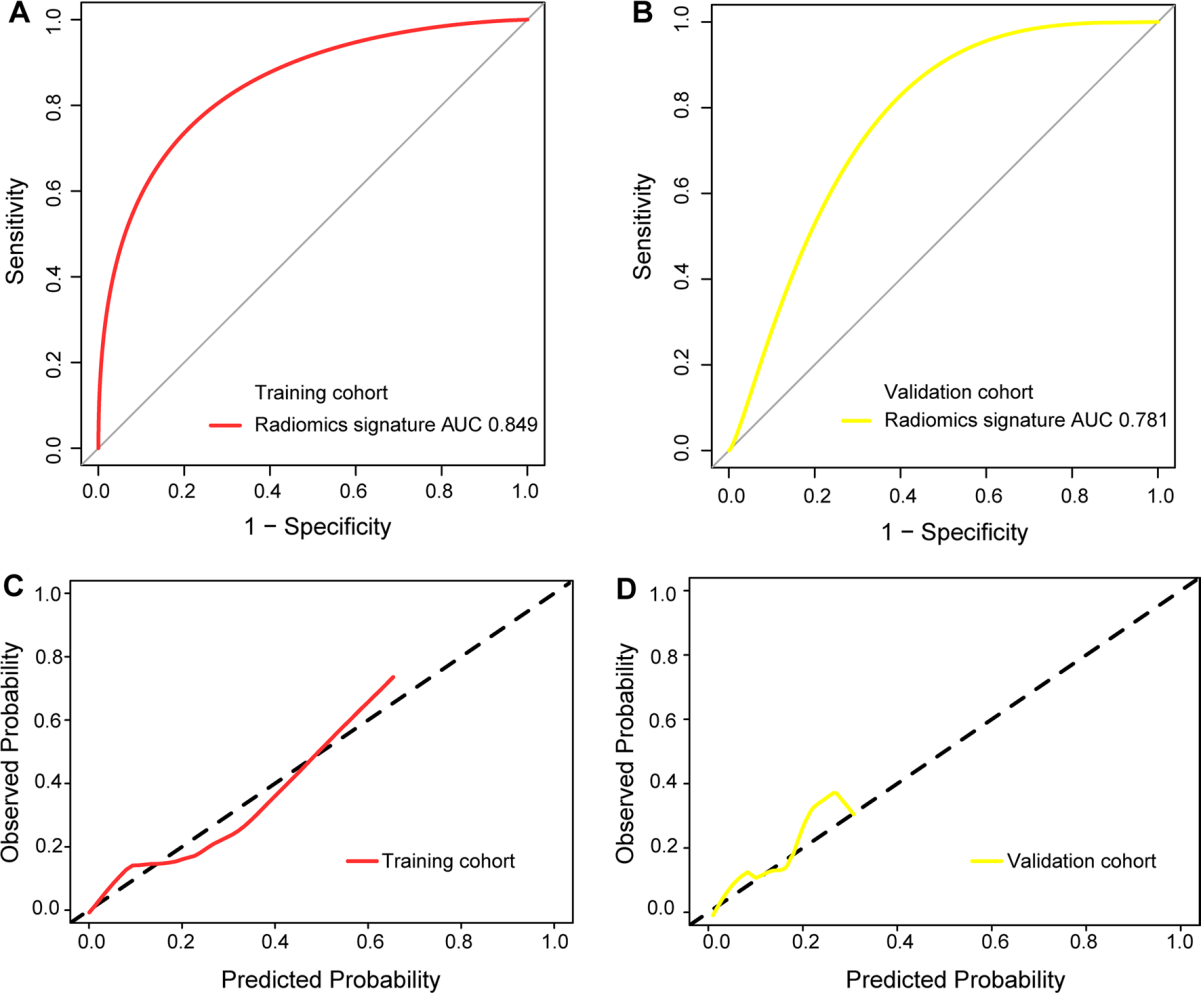
**a** AUC of the receiver operating characteristic (ROC) curve in the training set. **b** AUC of the ROC curve in the validation set. **c** Calibration curve in the training set. **d** Calibration curve in the validation set

**Fig. 7:**
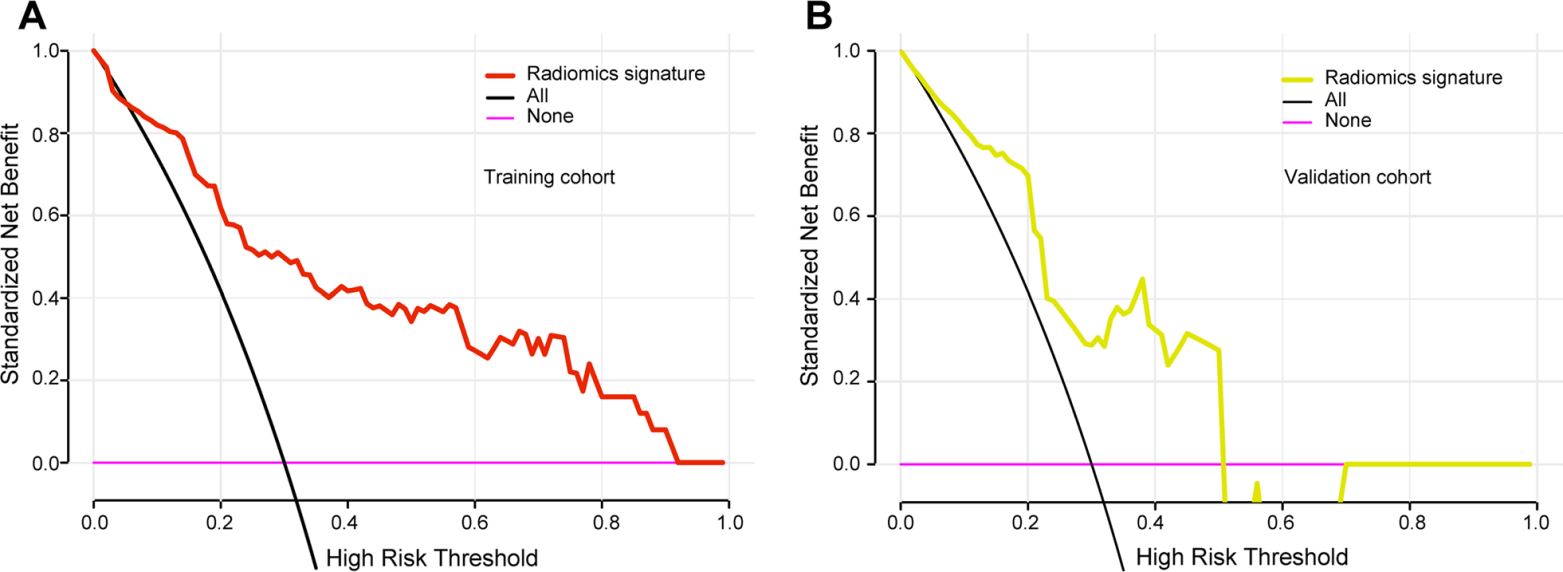
Predictive performance of the model and its decision curve analysis. **a**, **b** are the decision curves of the probabilistic relationship between net income and threshold drawn based on the training and verification sets, respectively. The probability of the X-axis high-risk threshold is the probability of LVI in this study, ranging from 0 to 1. The Y-axis measures net income. The black line shows that all rectal cancer patients are free of LVI, and the yield is 0. The purple curve indicates that LVI occurred in all patients

We used logistic regression on the optimal subset of features to build a radiomics model in this training set, and we also applied the validation set for model evaluation. The ROC curves (Fig. 6) showed that the radiomics model had good diagnostic efficacy in both sets (AUC of 0.849, sensitivity of 0.76, specificity of 0.75, and accuracy of 0.86 in the training set; AUC of 0.781, sensitivity of 0.75, specificity of 0.79, and accuracy of 0.83 in the validation set). The calibration curve suggested that the radiomics model in our study had good accuracy (Fig. 6). The results of the DCA showed that the radiomic features were clinically useful (Fig. 7).

## Discussion

RC has a high incidence and poor prognosis. Microvascular invasion is an important factor in the residual and recurrence of RC [26]. At this stage of clinical practice, microvascular invasion is mainly identified by postoperative pathology, with a certain lag. Our study purpose was to develop a preoperative prediction model for LVI of RC using endorectal ultrasound-based radiomics. The model showed good predictive value in both training and validation sets, with AUC of 0.849 and 0.781, respectively, suggesting that endorectal ultrasound-based radiomics may predict the vascular invasion of RC to provide a reference for clinical treatment decisions.

Radiomics is useful in the diagnosis, staging, treatment response, and prognosis of many solid tumors. For example, radiomics can be applied to the differential diagnosis of HCC [24], the assessment of the efficacy of neoadjuvant chemotherapy for breast cancer [27], and the characterization, grading, and prediction of tumor outcome in kidney cancer [28]. Radiomics also has important value in diagnostic staging [29], treatment response [23, 30], and prognosis of RC [21]. For LVI of RC, CT-based radiomics and MRI-based radiomics have good diagnostic efficacy for microvascular infiltration of RC [18, 19]. However, endorectal ultrasound-based radiomics has not been reported for vascular invasion in RC.

Ultrasound is an inexpensive, quick, and noninvasive examination, which reflects its worth in offering TNM stage information of RC. Patients may gain benefits if this examination combining radiomics is used to predict the LVI of RC noninvasively before resection.

There have been some studies of radiomics predicting vascular invasion in RC. Zhang et al. [19]. applied MR radiomics to predict LVI with an AUC of 0.884 in the training set and 0.876 in the validation set, but they included only 94 patients in this study, and this study extracted only 1188 radiomic features. A study using CT radiomics to predict RC LVI also extracted only 396 radiomic features [18]. Compared with the above two studies, our study had more patients and features, including a total of 203 patients and extracting 5936 radiomic features.

Nevertheless, radiomics has limitations, especially in its operator dependence on manual outlining of the ROIs and the generalizability of the model [31]. For example, Yao et al. [32]. predicted HCC subtypes, KI-67, and microvascular invasion (MVI) by using radiomics of multimodal ultrasound images; however, they only performed a model of the training set without the validation set and did not perform the ICC test. Li et al. [33] applied CT radiomics to develop a model for predicting distant metastasis in RC; in this study, the validation set was available, but the ICC test was not performed. The above studies did not perform ICC tests on the ROIs to assess the consistency between different operators and the reproducibility of these models. In our research, the prediction model is not only established by a training set but also verified by a validation set to ensure the universality of the model. At the same time, we performed an ICC test on the outlined portion of the ROIs to test the consistency between different operators. The results suggested that the ICC is above 0.75 in 90.1% (5350/5936), which guaranteed the reproducibility of our study, and that similar results can be obtained when the model is used by different analysts. Also, in the process of radiomics performance, we used CLAIM (Checklist for Artificial Intelligence in Medical Imaging) as the Radiomics Quality Score to make sure the methodological quality [34]. And this checklist was added in the Additional file 1.

This study has the following limitations. First, the case selection bias may exist in our retrospective study, which should be avoided by prospective studies in the future. Second, the ultrasound images used in this study were from different operators using different machines, and these confounding factors could potentially affect the predictive performance of the model, so the next step requires a prospective cohort study with strictly controlled variables (e.g., one operator, with one machine to acquire images). Third, ultrasound images only take the largest section for analysis. Although it can represent a lesion, it still cannot represent the whole lesion, and information may still be missed. Future studies need to analyze the overall lesions by forming three-dimensional images. Four, the limit number of cases is also one of our limitations. We need further research with large samples and multicenter data in the future.

## Conclusions

In summary, we have developed a prediction model for endorectal ultrasound-based radiomics, which is expected to be used to predict the presence of RC LVI. This method can be promoted in clinical practice because it can noninvasively predict the presence or absence of LVI before surgery and thus influence clinical decision-making.

## Supplementary Information

**Additional file 1:** CLAIM (Checklist for Artificial Intelligence in Medical Imaging).

## Data Availability

The datasets generated and analyzed during the current study are not publicly available due to the original datasets containing personal privacy information but are available from the corresponding author on reasonable request.
